# Genomic Epidemiology of a Protracted Hospital Outbreak Caused by a Toxin A-Negative Clostridium difficile Sublineage PCR Ribotype 017 Strain in London, England

**DOI:** 10.1128/JCM.00648-15

**Published:** 2015-09-16

**Authors:** M. D. Cairns, M. D. Preston, T. D. Lawley, T. G. Clark, R. A. Stabler, B. W. Wren

**Affiliations:** a Department of Pathogen Molecular Biology, London School of Hygiene and Tropical Medicine, London, United Kingdom; b UCL Centre for Clinical Microbiology, University College London, London, United Kingdom; c Public Health Laboratory London, Health Protection Agency, Division of Infection, The Royal London Hospital, London, United Kingdom; d Wellcome Trust Sanger Institute, Wellcome Trust Genome Campus, Hinxton, Cambridgeshire, United Kingdom; e Department of Infectious Disease Epidemiology, London School of Hygiene and Tropical Medicine, London, United Kingdom

## Abstract

Clostridium difficile remains the leading cause of nosocomial diarrhea worldwide, which is largely considered to be due to the production of two potent toxins: TcdA and TcdB. However, PCR ribotype (RT) 017, one of five clonal lineages of human virulent C. difficile, lacks TcdA expression but causes widespread disease. Whole-genome sequencing was applied to 35 isolates from hospitalized patients with C. difficile infection (CDI) and two environmental ward isolates in London, England. The phylogenetic analysis of single nucleotide polymorphisms (SNPs) revealed a clonal cluster of temporally variable isolates from a single hospital ward at University Hospital Lewisham (UHL) that were distinct from other London hospital isolates. *De novo* assembled genomes revealed a 49-kbp putative conjugative transposon exclusive to this hospital clonal cluster which would not be revealed by current typing methodologies. This study identified three sublineages of C. difficile RT017 that are circulating in London. Similar to the notorious RT027 lineage, which has caused global outbreaks of CDI since 2001, the lineage of toxin-defective RT017 strains appears to be continually evolving. By utilization of WGS technologies to identify SNPs and the evolution of clonal strains, the transmission of outbreaks caused by near-identical isolates can be retraced and identified.

## INTRODUCTION

Clostridium difficile causes a spectrum of disease ranging from mild diarrhea to life-threatening colitis, mainly in elderly, hospitalized patients. The disease pathogenesis is largely considered to be due to the production of two potent toxins: TcdA and TcdB (1).

The global emergence of the PCR ribotype (RT) 027 strain was responsible for multiple outbreaks and increased disease severity in Canada and the United States in 2001 (2). This strain has since spread to South America, China, Japan, Hong Kong, Korea, Singapore, Australia, and New Zealand and throughout Europe (3). Although RT027 remains the dominant clone in the United States, Europe has seen a decline in this RT. This has occurred simultaneously with an increase in other virulent RTs such as RT017 and RT078 (4–7).

The genome and phylogeny of RT027 have been well studied (2, 8–10), and multilocus sequence typing (MLST) and whole-genome sequencing (WGS) studies have confirmed the existence of at least five C. difficile clonal lineages where one is made up mostly of RT027 and another of RT017 (11). Pathogenic RT027 strains produce both toxins A and B and a third unrelated binary toxin (CDT) that has been implicated in virulence (12). In contrast, RT017 strains invariably lack most of the *tcdA* gene and completely lack the CDT gene yet have emerged worldwide, causing significant disease (6, 13). The reasons for the emergence of a less toxigenic lineage remain unclear. There is evidence that the prevalence of clinically relevant cases of CDI due to toxin A^−^B^+^ strains has increased globally (14–16). RT017 strains have been reported in The Netherlands (17), Poland (18), Ireland (14), China (19), Korea (20–22), Argentina (23), Australia (15), Israel (24), and Japan (25). Interestingly, the epidemiology of C. difficile in the Asia/Pacific regions and eastern parts of Europe appears to differ from that elsewhere where the prevalence of toxin A^−^B^+^ strains was higher than the prevalence other RTs, including RT027. Furthermore, given that some diagnostic laboratories rely on detecting toxin A, the incidence of A^−^B^+^ RT017 is likely to be significantly underreported.

C. difficile is a relatively clonal organism (8, 26, 27) and therefore amenable to WGS and subsequent single nucleotide polymorphism (SNP) analysis. WGS offers considerable advantages over traditional phenotypic and genotypic typing methods and performs a fine-grained analysis that facilitates the accurate tracing of the sources and routes of transmission (28).

The University Hospital Lewisham (UHL) in South London experienced multiple, temporally variable clusters of RT017 between March 2009 and April 2011 in one elderly care ward. This study investigates the genotypic characteristics of these clusters of 18 RT017 isolates and two environmental RT017 isolates from the ward, one community RT017 isolate from a patient who had spent time on the ward, two RT017 isolates from patients who spent time in other locations in UHL, and 13 human isolates from other London hospitals in a similar time period using WGS (Table 1).

**TABLE 1:** Study isolates

Isolate	Sample date (day/mo/yr)	Hospital [date(s) (day/mo/yr) patient in elderly care ward at UHL^*a*^]
H-UHL-1^*b*^	2005	UHL (ward nonexistent)
UHL-2	11/03/09	UHL (time of specimen)
UHL-3	27/03/09	UHL (24/02/09−05/03/09)
UHL-4	17/04/09	UHL (time of specimen)
UHL-5	16/04/09	UHL (time of specimen)
UHL-6	28/09/09	UHL (time of specimen)
UHL-7	20/09/09	UHL (time of specimen)
UHL-8	16/10/09	UHL (time of specimen)
UHL-9	29/10/09	UHL (time of specimen)
UHL-10	28/01/10	UHL (time of specimen)
UHL-11	08/02/10	UHL (time of specimen)
UHL-12	17/02/10	UHL (time of specimen)
UHL-13	01/04/10	UHL (time of specimen)
UHL-14	26/04/10	UHL (time of specimen)
UHL-15	17/07/10	UHL (time of specimen)
UHL-16	19/07/10	UHL (time of specimen)
UHL-17	06/08/10	UHL (never)
UHL-18	10/08/10	UHL (time of specimen)
E-UHL-19^*c*^	13/08/10	UHL (ward: side-room toilet)
E-UHL-20^*c*^	13/08/10	UHL (ward: side-room floor)
UHL-21	04/10/10	UHL (never)
UHL-22	07/10/10	UHL (04/06/10−12/07/10)
UHL-23	26/04/11	UHL (15/02/11−14/04/11)
C-UHL-24^*d*^	08/03/13	UHL (26/12/12−28/12/12, 04/02/13−07/02/13, and 08/03/13−20/02/13)
NP-25	13/05/08	Northwick Park Hospital
B-26	27/02/09	Barnet Hospital
NM-27	2005	North Middlesex Hospital
GOSH-28	22/03/10	Great Ormond Street Hospital
GOSH-29	24/03/10	Great Ormond Street Hospital
GOSH-30	27/03/10	Great Ormond Street Hospital
RF-31	09/12/10	Royal Free Hospital
CX-32	25/01/11	Charing Cross Hospital
B-33	11/05/11	Barnet Hospital
QM-34	16/07/11	Queen Mary's Hospital
WX-35	08/12/11	Whipp's Cross Hospital
WX-36	16/01/12	Whipp's Cross Hospital
GOSH-37	30/11/13	Great Ormond Street Hospital

a UHL, University Hospital Lewisham.

b Historical isolate predating the building of the hospital ward.

c Environmental isolate recovered from the ward.

d Community-acquired infection isolate.

## MATERIALS AND METHODS

### Bacterial study isolates and growth conditions.

Study isolates are shown in Table 1 (European Nucleotide Archive study accession number ERP009770). Thirteen isolates from other London hospitals were also included to place these RT017 A^−^B^+^ C. difficile cluster isolates from UHL into a geographical and historical context. The RT017 strain M68, which has been fully sequenced, was isolated from an outbreak that affected two hospitals in Dublin (Mater Misericordiae and St Vincent's University Hospitals) in Ireland in 2006. This was used as the control RT017 strain for genomic comparisons (GenBank accession number FN668375).

C. difficile isolates were routinely cultured at 37°C under anaerobic conditions (Don Whitley Scientific, West Yorkshire, United Kingdom) using blood agar (Oxoid, Hampshire, United Kingdom) and then brain heart infusion broth (BHI) (Oxoid) plus C. difficile supplement (Sigma, Dorset, United Kingdom). Broths were incubated on a shaking platform at 60 rpm.

### DNA extraction and whole-genome sequencing.

Genomic DNA was extracted as previously described by Stabler et al. (9). WGS data for the isolates were obtained using either the HiSeq 2000 sequencing system or the MiSeq sequencing system (Illumina, CA, USA). Libraries were created as previously described (29) or using a Nextera XT kit (Illumina, CA, USA), respectively. Minimum and maximum numbers of paired reads of 910,000 and 2,848,000, respectively, were generated.

### Whole-genome bioinformatic analysis.

The sequence data were processed according to a standard pipeline as previously described (30). Briefly, FASTQ-formatted sequencing reads were quality controlled with a minimum-quality Phred score of 30 (as a rolling average over 4 bases) using Trimmomatic (31). The resulting reads were mapped, using BWA-MEM software (32), against the M68 C. difficile reference strain (4,308,325 bp). The majority of posttrimmed reads were mapped to the reference (median, 98.1%; range 94.3% to 99.8%) with a mean depth of 64.6 bp per sample (range, 38.3 bp to 114.2 bp) and median 61,000 loci with no base call made (1.4% of the entire genome; range, 0.4% to 3%). SNP loci with a minimum quality score of 30 were identified using SAMtools (33). SNP mutations in the samples that had read depths of >60 and had >70% of reads identified with the same allele (99.8% of SNPs were supported by >90% of contributing reads) were identified. In total, 748 SNPs were identified at 162 biallelic SNP loci: 94 (58.0%) loci coded for nonsynonymous genic changes, 21 (13.0%) loci encoded for synonymous genic changes, and 47 (29.0%) loci were in nongenic change regions. In addition, Velvet (34) and VelvetOptimiser (35) were used to *de novo* assemble the trimmed reads into contigs. Thirty-five high-quality assemblies were produced. Optimal k-mers fell between 63 and 81 bp. The mean *N*_50_ was >1 Mbp (range, 89 kbp to 4.2 Mbp). The mean longest contig was 1,122,300 bp (including 3 samples with >4 Mbp [96%] of the genome in a single contig). The two remaining assemblies produced contigs that equated to >97% of the genome but were highly fragmented for all postanalyses. Pipeline analysis, postanalyses, and genetic and phylogenetic analysis were carried out using Perl, R, ABACAS, Prokka, and RAxML software (36–39).

## RESULTS

All 37 isolates were confirmed to be RT017 by repeat PCR ribotyping. Eight isolates from the UHL temporal clusters were subtyped using multilocus variable-number tandem-repeat analysis (MLVA): six were found to be indistinguishable (UHL-2, UHL-4, UHL-5, UHL-6, UHL-7, and UHL-9) and two differed from the other six isolates by only one locus (UHL-3 and UHL-8).

### Whole-genome SNP and *de novo* genome assembly analysis.

The 37 isolates were whole-genome sequenced, and the resulting data were processed to identify and analyze high-quality SNPs (30). A total of 162 biallelic SNP loci in the samples from the 4,308,325 bp of the M68 reference were identified; the majority (79.0% [128/162]) exhibited a minor allele frequency (MAF) of <10%, including 54.3% (88/162) of loci being identified in one sample. Only 17 SNP loci (10.5%) had a nonreference allele frequency of >50%. Each isolate contained up to 46/162 (28.4%) mutations, with 64.9% (24/37) of isolates containing between 17 and 19 (10.5 to 11.7%) SNPs.

**FIG 1:**
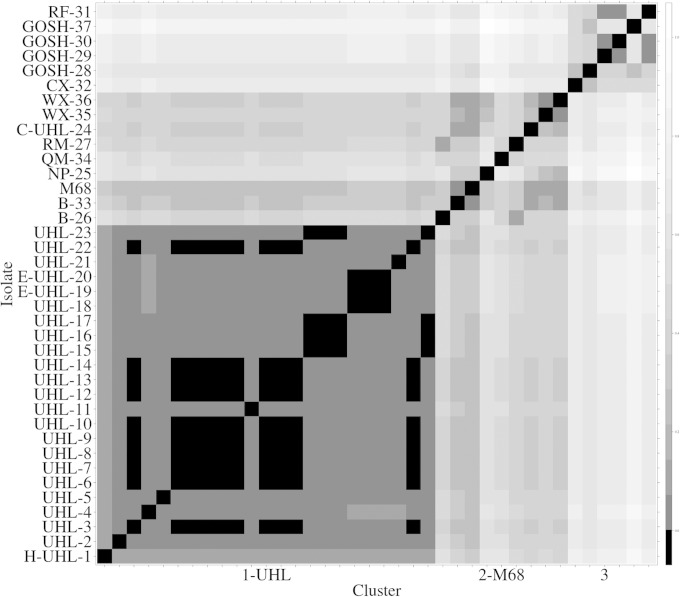
A heat map showing inter- and intracluster relatedness. A normalized Manhattan distance between samples was used, with darker shades indicating a closer genetic identity between sample pairs.

**FIG 2:**
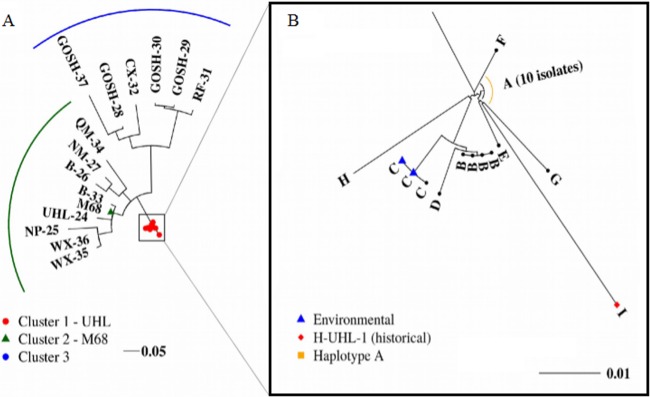
(A) Maximum-likelihood phylogenetic analysis of all 37 London isolates based on core genome SNPs against the M68 reference. (B) Cluster 1-UHL haplotypes.

The data set revealed 23 different SNP patterns (haplotypes), 9 of which (labeled A to I) were only found in isolates H-UHL-1 and UHL-2 to UHL-23 (Tables 2 and 3; Fig. 1). There are 24 SNP loci unique to these 23 UHL samples (16 nonsynonymous, 5 synonymous, and 3 nongenic). Haplotype A is the core haplotype with only the 11 SNPs common to all of these UHL isolates. Haplotypes B to H differ from haplotype A by 1 or 2 extra SNPs. Haplotype I was distinguishable from haplotype A by 5 SNPs. A heat map of the genetic Manhattan distance (Fig. 1) and a maximum-likelihood phylogenetic tree (generated by RAxML [39]) (Fig. 2A) revealed three related groups of samples. Cluster 1-UHL was composed exclusively of haplotypes A to I, containing 23 (out of 24) isolates from UHL (Fig. 2B). Cluster 2-M68 (containing the M68 reference strain) encompasses the outer London hospitals and a UHL patient (UHL-24) who had community-acquired CDI. Cluster 3 contains all of the samples from the three inner London hospitals (GOSH, Royal Free, and Charing Cross). Twenty-four (14.8%), 61 (37.7%), and 71 (43.8%) SNP loci are found exclusively in clusters 1-UHL, 2-M68, and 3, respectively, and only six SNP loci (3.8%) have mutations in more than one cluster. Of these loci exclusive to the three clusters, 11 (out of 24 SNP loci), 1 (out of 61), and 16 (out of 72) were found in every sample in their respective clusters.

**TABLE 2:** SNPs unique to cluster 1-UHL (isolates H-UHL-1 and UHL-2 to UHL-23 inclusive)

Position	Reference residue	Alternative residue	Type	Product/putative function	Haplotype
345335	S	R	Nonsynonymous	Protein-tyrosine phosphatase reductase	A to I
433205	S	S	Synonymous	Formate/nitrite transporter	A to I
578215	P	S	Nonsynonymous	Iron hydrogenase	A to I
707105	F	L	Nonsynonymous	Multidrug family ABC transporter permease	A to I
1123155	G	S	Nonsynonymous	Putative membrane protein	A to I
1241002	L	L	Synonymous	NhaC family Na^+^/H^+^ antiporter	A to I
1316457	A	A	Synonymous	3-Hydroxybutyrate dehydrogenase	A to I
2764775	P	L	Nonsynonymous	Diguanylate kinase signaling protein	A to I
3072208	G	A	Nonsynonymous	Maf-like protein	A to I
3202066	L	F	Nonsynonymous	Multidrug family ABC transporter	A to I
4025381			Intergenic	Unknown	A to I
1491685			Intergenic	Unknown	B, C, and D
584197	C	R	Nonsynonymous	Response regulator (quorum-sensing system)	E
1245898	H	N	Nonsynonymous	Copper-sensing transcriptional repressor CsoR	H
1395682	I	L	Nonsynonymous	Hypothetical protein	H
583796	R	L	Nonsynonymous	Response regulator (quorum-sensing system)	F
1932695	I	V	Nonsynonymous	Putative membrane protein	C
3698806			Intergenic	Unknown	G
3056134	L	I	Nonsynonymous	RNase G	D
34552	S	Y	Nonsynonymous	DNA-directed RNA polymerase beta chain	I
2744067	E	E	Synonymous	Putative TPR repeat-containing protein	I
2813984	E	E	Synonymous	GntR family transcriptional regulator	I
3289962	S	Y	Nonsynonymous	ABC transporter substrate-binding protein	I
3766047	K	Stop codon	Nonsynonymous	Two-component response regulator	I

**TABLE 3:** Haplotypes

Haplotype	SNP position in M68 reference (at bp)^*a*^																							
	34552	345335	433205	578215	583796	584197	707105	1123155	1241002	1245898	1316457	1395682	1491685	1932695	2744067	2764775	2813984	3056134	3072208	3202066	3289962	3698806	3766047	4025381
A		✓	✓	✓			✓	✓	✓		✓					✓			✓	✓				✓
B		✓	✓	✓			✓	✓	✓		✓		✓			✓			✓	✓				✓
C		✓	✓	✓			✓	✓	✓		✓		✓	✓		✓			✓	✓				✓
D		✓	✓	✓			✓	✓	✓		✓		✓			✓		✓	✓	✓				✓
E		✓	✓	✓		✓	✓	✓	✓		✓					✓			✓	✓				✓
F		✓	✓	✓	✓		✓	✓	✓		✓					✓			✓	✓				✓
G		✓	✓	✓			✓	✓	✓		✓					✓			✓	✓		✓		✓
H		✓	✓	✓			✓	✓	✓	✓	✓	✓				✓			✓	✓				✓
I	✓	✓	✓	✓			✓	✓	✓		✓				✓	✓	✓		✓	✓	✓		✓	✓

a The presence of an SNP is depicted by a checkmark.

**FIG 3:**
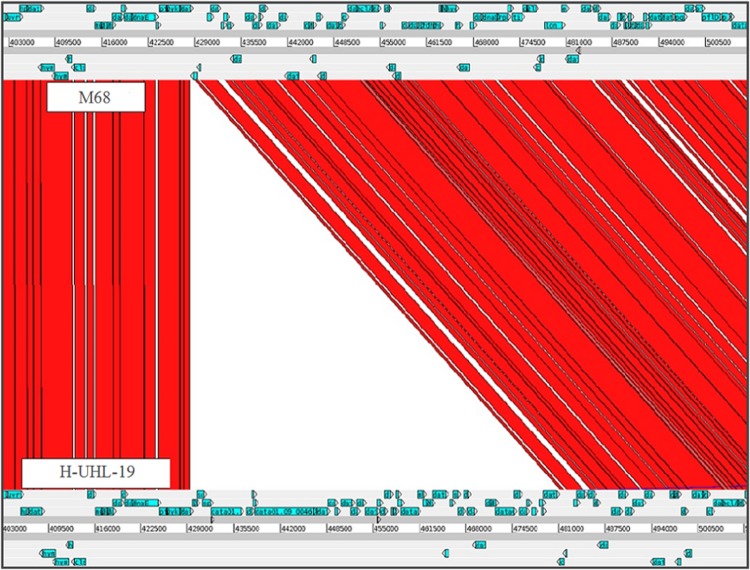
Artemis Comparison Tool (ACT) comparison between C. difficile strains M68 and H-UHL-19, illustrating the putative chromosomal transposon exclusive to H-UHL-19 and other members of the cluster 1-UHL.

*De novo* assembly of each isolate and comparison to the M68 reference strain revealed a 49-kbp, chromosomal, genetic region exclusive to cluster 1-UHL (23/37 isolates) (Fig. 3). Programmatic and visual inspection of the comparisons revealed no other large structural variations between samples. Using Prokka (38) to annotate this 49-kbp putative transposon with predicted protein coding regions, we identified 45 sequences that have an ortholog to known gene sequences (see Table S1 in the supplemental material). Predicted genes include sortase (sortase B), sporulation (Spo0J), and collagen-binding proteins with known links to phenotypic and virulence markers. These 45 predicted genes are highly conserved with 41 being amino-acid identical across all 23 samples in cluster 1-UHL.

## DISCUSSION

Between 2009 and 2011, an elderly care ward at UHL experienced multiple, temporally variable clusters of CDI caused by RT017. This study analyzed the genotypic characteristics of these 20 RT017 isolates along with a further 17 RT017 isolates: 2 isolates from patients nursed in other wards in UHL, 1 community-acquired isolate, 1 historical isolate from UHL that predates the building of the hospital ward, and 12 isolates from other London hospitals.

Phylogenetic analysis of the SNPs identified through WGS of the isolates revealed three related groups. Twenty-three UHL isolates (including the historical H-UHL isolate) formed a tight cluster with only nine haplotypes and few SNP differences between them. The 13 non-UHL isolates, the community-acquired isolate (C-UHL-24), and the reference M68 formed two additional clusters shown in Fig. 2A: cluster 2-M68 and cluster 3. A highly conserved putative 49-kbp conjugate transposon was found exclusively in the assembled chromosomes of the 23 cluster 1-UHL isolates (Fig. 3). In combining these data, three diverging sublineages of RT017 are identified, with 96.3% of the SNP loci found exclusively in only one cluster and a large insertion further differentiating cluster 1-UHL.

In cluster 1-UHL, H-UHL-1 (haplotype I) is a historical isolate from 2005 which predates the other 22 isolates in the cluster and even the existing building where the elderly care ward is located. The H-UHL-1 sample/haplotype I was distinguishable from the UHL core haplotype (A) by only 5 SNPs. A study by Eyre et al. estimated an evolutionary rate of 0.74 SNP per year for C. difficile (40), meaning that the 4-year isolation gap between the historical isolate in 2005 and the first cluster 1-UHL isolate in 2009 is consistent with these mutation rates and suggests that the historical H-UHL-1 isolate shares a common ancestor with the cluster 1-UHL isolates. Haplotypes A to H were indistinguishable based on a ≤2 SNP difference, suggesting that they are the same strain and transmission has occurred between patients and/or via ward contamination. Additionally, the two environmental isolates (E-UHL-19 and E-UHL-20) recovered from the toilet and floor of the elderly care ward side room were indistinguishable from the cluster 1-UHL isolates; the environment was therefore contaminated with this strain. Two UHL isolates (UHL-17 and UHL-21) from patients who were never admitted to the elderly care ward were found to be part of cluster 1-UHL, strongly suggesting interward transmission. One isolate (C-UHL-24) recovered from a patient specimen taken on the day of admittance to the elderly care ward was distinguishable from the cluster 1-UHL isolates; however, this CDI was defined as community acquired with symptoms occurring ≤48 h after hospital admission.

On 30 September 2010, hydrogen peroxide vapor (HPV) decontamination was performed in the elderly care ward at UHL. Subsequent to this, three isolates of RT017 indistinguishable from the cluster 1-UHL isolates were recovered from patients in October 2010 and April 2011 (Table 1). This strain has persisted at UHL with a possible internal reservoir that was never eliminated during the HPV decontamination. With the historical isolate from 2005 being indistinguishable from the more recent clonal isolates, the possibility of an external source that has reintroduced this strain to the elderly care ward cannot be excluded.

These data suggest that a clonal strain has persisted in one London hospital for at least 5 years and is different from other RT017 strains that are circulating in London hospitals. Our study is a snapshot of isolates from hospitalized patients with CDI from London, England, and demonstrates the persistence of a toxin A^−^B^+^ RT017 strain in a ward at UHL since at least 2005. We identify three lineages, one harboring a large putative conjugate transposon found exclusively in the cluster 1-UHL isolates.

This lineage of toxin A- and CDT-defective RT017 strains appears to be evolving similar to the RT027 counterpart, which has caused global outbreaks since 2001. The RT017 lineage, with its unique toxin profile and unusual global prevalence, is understudied. Our data show that there are existing questions about the biology, population structure, and epidemiology of toxin A^−^B^+^ RT017. Answering these will contribute to our understanding of the evolution of C. difficile across all lineages and help the diagnosis and treatment of CDI. With utilization of WGS technologies to identify SNPs and the evolution of clonal strains, the transmission of outbreaks caused by near-identical isolates can be retraced and identified.

## Supplementary Material

Supplemental material
