# Supplementary material for: Genomic Epidemiology of a Protracted Hospital Outbreak Caused by a Toxin A-Negative Clostridium difficile Sublineage PCR Ribotype 017 Strain in London, England
Source: J Clin Microbiol. 2015 Sep 16;53(10):3141–7. doi: 10.1128/JCM.00648-15 (PMC4572532; doi:10.1128/JCM.00648-15)
Supplement: Supplemental material [file supp_53_10_3141__index.html]

Genomic Epidemiology of a Protracted Hospital Outbreak Caused by a Toxin A-Negative Clostridium difficile Sublineage PCR Ribotype 017 Strain in London, England — Supplemental material 

# Genomic Epidemiology of a Protracted Hospital Outbreak Caused by a Toxin A-Negative Clostridium difficile Sublineage PCR Ribotype 017 Strain in London, England

## Supplemental material

- Supplemental file 1 -

  Table S1 (Predicted coding sequences from the putative chromosomal transposon exclusive to the cluster 1-UHL)

  PDF, 169K
