# Supplementary material for: Genomic Epidemiology of a Protracted Hospital Outbreak Caused by a Toxin A-Negative Clostridium difficile Sublineage PCR Ribotype 017 Strain in London, England
Source: J Clin Microbiol. 2015 Sep 16;53(10):3141–7. doi: 10.1128/JCM.00648-15 (PMC4572532; doi:10.1128/JCM.00648-15)
Supplement: Supplemental material [file JCM.00648-15_zjm999094492so1.pdf]

Supplementary Table 1: Predicted coding sequences from the putative chromosomal transposon exclusive to the cluster 1-UHL.

UHL-19	Putative Product/Role/Function according to NCBI Blast and UniProt
Data01.09_00455	Uncharacterised protein
Data01.09_00456	Uncharacterised protein
Data01.09_00457	Soj (parA) protein (plasmid and chromosome partitioning)
Data01.09_00458	Spo0J (parB) protein (plasmid and chromosome partitioning)
Data01.09_00459	Collagen-binding Cna protein
Data01.09_00460	Uncharacterised protein
Data01.09_00461	RadA - ATP-dependent protein (DNA repair and degradation of proteins)
Data01.09_00462	Uncharacterised protein
Data01.09_00463	Superfamily II DNA and RNA helicase (RNA and DNA metabolism)
Data01.09_00464	Uncharacterised protein
Data01.09_00465	None
Data01.09_00466	Uncharacterised protein
Data01.09_00467	Uncharacterised protein
Data01.09_00468	Endonuclease relaxase (horizontal transfer of plasmid genetic information)
Data01.09_00469	Uncharacterised protein
Data01.09_00470	Uncharacterised protein
Data01.09_00471	Conjugative transfer of transposon-like mobile genetic elements, Type IV
Data01.09_00472	Aspartyl/glutamyl – tRNA (Asn/Gln) (carbon-nitrogen ligase activity)
Data01.09_00473	Adenine-specific methyltransferase (macrolide resistance)
Data01.09_00474	Uncharacterised protein
Data01.09_00475	Phd_YefM (antitoxins in type II toxin-antitoxin systems)
Data01.09_00476	Plasmid stabilisation system protein
Data01.09_00477	Uncharacterised protein
Data01.09_00478	PrgI family protein (uncharacterised protein)
Data01.09_00479	AAA-like domain protein (uncharacterised protein)
Data01.09_00480	Modification methylase MboII (DNA methylation)
Data01.09_00481	Uncharacterised protein
Data01.09_00482	Sortase B (surface protein attachment)
Data01.09_00483	Transglycosylase (enzymatic activity)
Data01.09_00484	MerR family transcriptional regulator (DNA binding)
Data01.09_00485	Uncharacterised protein
Data01.09_00486	Uncharacterised protein
Data01.09_00487	Conjugative transposon protein (enzymatic activity)
Data01.09_00488	Tetracycline resistance, transcriptional regulator
Data01.09_00489	Iron-sulfur protein (4Fe-4S binding domain)
Data01.09_00490	Multi antimicrobial extrusion protein (drug transportation)

Data01.09_00491	DNA binding, transcriptional regulator
Data01.09_00492	ABC transporter family protein (membrane transport)
Data01.09_00493	ftsX-like permease family protein (membrane structure)
Data01.09_00494	Two-component response regulator (uncharacterised protein)
Data01.09_00495	Two-component sensor histidine kinase (environmental stimuli response)
Data01.09_00496	Cysteine-rich KTR (uncharacterised protein)
Data01.09_00497	RNA polymerase ECF-type sigma factor (RNA transcription)
Data01.09_00498	PemK family transcriptional regulator (cell growth regulation)
Data01.09_00499	Short C-terminal (SHOCT) domain (oligomerisation and nucleic acid binding)
